# Short-Term Effects of PM_10_ and NO_2_ on Respiratory Health among Children with Asthma or Asthma-like Symptoms: A Systematic Review and Meta-Analysis

**DOI:** 10.1289/ehp.0900844

**Published:** 2009-11-12

**Authors:** Gudrun Weinmayr, Elisa Romeo, Manuela De Sario, Stephan K. Weiland, Francesco Forastiere

**Affiliations:** 1 Institute of Epidemiology, Ulm University, Ulm, Germany; 2 Department of Epidemiology, Local Health Authority Rome E, Rome, Italy

**Keywords:** air pollution, asthma, children, NO_2_, PM, short-term effects

## Abstract

**Objective:**

Our goal was to quantify the short-term effects of particulate matter with aerodynamic diameter ≤ 10 μm (PM_10_) and nitrogen dioxide (NO_2_) on respiratory health of asthmatic children from published panel studies, and to investigate the influence of study and population characteristics as effect modifiers.

**Data extraction:**

After a systematic literature review, we extracted quantitative estimates of the association of PM_10_ and/or NO_2_ with respiratory symptoms and peak expiratory flow (PEF). Combined effect estimates for an increase of 10 μg/m^3^ were calculated by random effects meta-analysis for all studies and for different strata defined by study characteristics. The effect of publication bias was investigated with Egger’s and Begg’s tests and “trim-and-fill” analyses.

**Data synthesis:**

We identified 36 studies; 14 were part of the European Pollution Effects on Asthmatic Children in Europe (PEACE) study. Adverse associations of PM_10_ with asthma symptoms were statistically significant [odds ratio (OR) = 1.028; 95% confidence interval (CI), 1.006–1.051]. There were also associations, although not statistically significant, of PM_10_ with cough (OR = 1.012; 95% CI, 0.997–1.026) and on PEF (decrease of −0.082 L/min; 95% CI, −0.214 to 0.050). NO_2_ had statistically significant associations with asthma symptoms in the overall analysis considering all possible lags (OR = 1.031; 95% CI, 1.001–1.062), but not when we evaluated only the 0–1 lag. We found no publication bias, although it appeared when excluding the PEACE studies. When we applied the trim-and-fill method to the data set without the PEACE studies, the results were similar to the overall estimates from all studies. There was an indication for stronger PM_10_ associations for studies conducted in summer, outside of Europe, with longer lags, and in locations with higher NO_2_ concentrations.

**Conclusions:**

We found clear evidence of effects of PM_10_ on the occurrence of asthma symptom episodes, and to a lesser extent on cough and PEF. The results for NO_2_ are more difficult to interpret because they depend on the lag times examined. There was an indication of effect modification by several study conditions.

Particulate matter (PM) with an aerodynamic diameter ≤ 10 μm (PM_10_) and nitrogen dioxide (NO_2_) are important ambient air pollutants regulated by European and national legislations. Measurements of PM_10_ include PM of different aerodynamic diameter (coarse, fine, and ultrafine PM), and the size distribution is related to the emission source, with the coarse fraction mainly originating from soil and natural sources and fine and ultrafine PM mainly originating from combustion or being secondary aerosols from sources that can be far away (Williams 1999). Notwithstanding possible long-range transport, most of NO_2_ in the ambient air arises from oxidization of emitted NO*_x_* from combustion mainly from motor engines in urban areas (Williams 1999), and it is considered to be a good marker of traffic-related air pollution.

The health effects of PM_10_ and NO_2_ have been extensively reviewed, and air quality standards and guidelines have been proposed to protect public health [U.S. Environmental Protection Agency (EPA) 2005, 2008a; World Health Organization (WHO) Regional Office for Europe 2000, 2006]. Nevertheless, important clinical effects are currently detectable in real-life exposure to traffic-related pollutants among susceptible subgroups of the population, such as individuals with asthma. A recent study from London has clearly shown that asthmatic adults have a significant decrease in lung function after 2 hr of walking along a street in the center of London as opposed to walking in a nearby park (McCreanor et al. 2007). The effects were stronger in individuals with moderate asthma compared with individuals with mild asthma. Several studies have been conducted among children with asthma focusing on the short-term effects of air pollution, that is, its effects on daily symptoms and lung function. Most studies used air pollution measurements from central monitoring sites that provide daily data. Mostly PM_10_, NO_2_, and ozone (O_3_) have been evaluated; results for carbon monoxide, black smoke, and PM_2.5_ (PM with aerodynamic diamter ≤ 2.5 μm) have been less reported to date. Studies on long-term effects typically involve proximity of the residence to roads, but they do not provide information on short temporal scales.

Both PM_10_ and NO_2_ have been associated with increases in the frequency of asthma symptoms and with lung function decrements in children on a day-to-day scale (Gielen et al. 1997; Ostro et al. 2001; Pope and Dockery 1992; Roemer et al. 1993; Romieu et al. 1996; Schildcrout et al. 2006; van der Zee et al. 1999; Vedal et al. 1998). However, the results of the existing studies have not been consistent, and a comprehensive quantitative evaluation of the respiratory effect in children is still lacking.

Two meta-analyses on the short-term effects of PM_10_ on children’s respiratory health have previously been performed (Anderson et al. 2004; Ward and Ayres 2004). Anderson et al. (2004) reviewed the effects on cough and medication use in European panel studies, a large number of which were conducted within the multicenter PEACE (Pollution Effects on Asthmatic Children in Europe) study that provided 28 of the 34 effect estimates. In their review, they found no effect of PM_10_ on cough in children [odds ratio (OR) = 0.999 for 10-μg/m^3^ increase in PM_10_; 95% confidence interval (CI), 0.987–1.011]. Ward and Ayres (2004) performed a meta-analysis of worldwide panel studies published through 2002 that included asthmatic and healthy children. They found a significant effect of PM_10_ on cough (OR = 1.004 per unit μg/m^3^ increase PM_10_; 95% CI, 1.002–1.006), on lower respiratory symptoms (LRS) or wheeze (OR = 1.004 per 1 μg/m^3^ PM_10_; 95% CI, 1.002–1.005), and on peak expiratory flow (PEF) (a decrease of −0.033 L/min per 1 μg/m^3^ in PM_10_; 95% CI, −0.019 to −0.047). In both meta-analyses, the results of the large multicenter European PEACE study had a strong influence because of its primarily null results.

To our knowledge, no quantitative meta-analysis on the effects of NO_2_ among children with asthma has so far been performed. The available evidence is inconsistent, with some studies showing a detrimental effect of NO_2_ on symptoms or lung function and other investigations indicating no effect (Ackermann-Liebrich and Rapp 1999).

To provide a quantitative estimate of the acute effects of short-term exposure to PM_10_ and NO_2_ on respiratory symptoms and lung function in asthmatic children, we performed a meta-analysis on panel studies published through July 2008. We assessed the role of the PEACE study on the overall evaluation, and we paid specific attention to the influence of publication bias. Because study characteristics and pollution mixtures vary with space and time, some heterogeneity among the study results conducted at different locations is to be expected. We therefore investigated the influence of study and population characteristics on the outcomes.

## Methods

We conducted a systematic search of the literature from 1990 through July 2008 that focused on the short-term effects of outdoor NO_2_ and PM_10_ on respiratory health outcomes as determined in panel studies. To focus our study, we did not consider exposure to O_3_ or studies on indoor exposure; the latter has been typically investigated for long-term effects. We investigated lung function as measured by PEF and symptoms of cough and asthma, the latter being reported as wheeze or LRS. A MEDLINE (National Library of Medicine 2008) search was carried out; the search strings consisted of “asthma OR wheeze OR cough OR bronchitis OR lung function,” “air AND pollut*,” and “PM_10_ OR PM(10)” and “NO2 OR “NO(2)” OR “nitrogen dioxide.” Limits were set to retrieve only children (“All Child 0–18 years”). The exact search history is available from the authors. These criteria were applied to maximize sensitivity and to not miss any relevant publication. The age range of children in the panel studies was 5–19 years. Wheezing among infants was not considered because the asthma phenotype differs in very young children and there are essentially no panel studies on infants.

The references were then selected by hand according to the following inclusion/exclusion criteria: exclusion of indoor and laboratory studies; inclusion of panel studies on asthmatic or symptomatic (see definition below) children that reported a quantitative effect (regression coefficients); inclusion of only one publication of the same study/database for each outcome. With regard to the statistical analysis, we included only studies that controlled for the effect of daily temperature and day of the week, because these are important confounders and should be adjusted for to detect short-term effects of air pollution.

For the definition of “asthmatics” or “symptomatic” children, we relied on the criteria reported in the individual publications. Generally, children with asthma confirmed by a physician or who were referred from clinics, school nurses, and so on, with an asthma diagnosis were classified as “asthmatics.” We considered “symptomatic” children who reported, mostly in a questionnaire, wheezing or cough apart from cold or an asthma diagnosis, or who took medication for asthma.

The evaluated outcomes were “asthma symptoms” and “cough,” and the definitions differed in various studies, as indicated in “Results.” For PEF, we included only studies that reported changes as liters per minute or that allowed us to calculate the changes in liters per minute from the given percentages and were therefore directly comparable. Other lung function parameters and exhaled nitrogen oxide were not considered, because these studies are relatively scarce.

For the meta-analysis, we used the coefficients derived from single-pollutant models. Where necessary, the coefficient estimates were recalculated to reflect a 10-μg/m^3^ increase in pollutant assuming a linear relationship over the considered range. When coefficients for different lag times were given, we used the one that resulted in a statistically significant effect or, when all estimates were either significant or not significant, the lag reflecting the highest effect size. The same criterion was applied if lung function measurements were performed in the morning and in the evening. These criteria were modified in a sensitivity analysis as indicated below.

Combined estimates of the natural logarithm of the OR for respiratory symptoms and the linear regression coefficients for PEF, respectively, were calculated for all studies with a fixed effects and a random effects meta-analysis model (DerSimonian and Laird 1986; Petitti 2001) using the meta command of STATA (releases 8 and 9.1; StataCorp., College Station, TX, USA). This command uses inverse-variance weighting to calculate combined estimates. Although a fixed-effects model assumes that the studies reflect the same underlying average effect, in a random-effects model the study effects are coming from a common underlying distribution of effects. The corresponding weights include an additional term that reflects the between-study heterogeneity due to unexplained sources. Heterogeneity was assessed by calculating the *I*^2^ of Higgins and Thompson, which reflects the proportion of total variation in the combined estimate that is due to heterogeneity between studies (Higgins and Thompson 2002).

We evaluated publication bias with both the Begg test and the Egger test (Begg and Mazumdar 1994; Egger et al. 1997). The Egger et al. regression asymmetry test tends to suggest the presence of publication bias more frequently than the Begg adjusted rank correlation test, which has a low power.

Where necessary, a trim-and-fill analysis was performed to take account of publication bias (Duval 2000). This procedure estimates the number and outcomes of theoretical missing studies and incorporates them into the meta-analysis. All the calculations were done using the metabias and metatrim commands in STATA.

To explore heterogeneity in meta-analysis estimates, we considered the influence of the following study characteristics on meta-analytical estimates: continent (Europe; other countries), season (summer only; any other cases), population [asthmatics (confirmed diagnosis); symptomatics], duration (≤ 2 or > 2 months), lag (≤ 2 or > 2 days), average PM_10_ levels (< 40 or ≥ 40 μg/m^3^), and average NO_2_ levels (< 40 or ≥ 40 μg/m^3^). The influence of study characteristics was investigated by calculating the combined effect for each stratum and evaluating the difference between strata-specific estimates. The null hypothesis that the difference between the estimates from the two strata equals 0 was tested (with *Z*-score), and the corresponding *p*-value is reported here. Statistical significance was defined as *p* < 0.05 for all analyses.

Because the choice of the lag was a critical step, we performed additional analyses using, for all the studies, the effect estimate at lag 0–1 (instead of the most significant lag). The following criteria were applied. The default was lag 1; if lag 1 was not available, lag 0 or lag 0–1 was considered instead. In addition, we calculated the combined effects for PEF using only the evening values.

## Results

We retrieved a total of 77 references for PM_10_ and 324 for NO_2_. Applying the inclusion/exclusion criteria outlined in “Materials and Methods,” 36 studies on PM_10_ and 24 on NO_2_ remained to be included in the meta-analysis (Table 1). Some of the excluded studies were on indoor NO_2_, notably related with cooking and heating. Other studies were time-series analyses on hospital admissions, and a few studies were on pathologic mechanisms and exposure assessment. Of the total of 36 studies (on 51 populations), 14 were PEACE studies (28 populations). In this review, we refer to each population as a separate study and use the corresponding estimates. Peacock et al. (2003) studied a subgroup of wheezy children but did not give estimates for the coefficient for this group. Nevertheless, because the authors stated that there was no effect modification by wheeze, we took the estimate for all children instead.

Of the total of 51 populations studied, 36 were from Europe and 15 from elsewhere, mainly the United States. Thirty populations were from urban areas, and 20 studies were conducted in rural environments (one unspecified). Four studies were carried out in the summer only; the other studies were conducted mainly in winter or during most of the year. The mean 24-hr average for NO_2_ ranged from 8 to 77 μg/m^3^, and the mean 24-hr average for PM_10_ ranged from 11 to 167 μg/m^3^ (but only Mexico City had a value of 167 μg/m^3^; all the others had a value < 100 μg/m^3^).

The definition of the outcome regarding asthma symptoms varied among the studies: We included the estimates for wheeze from five studies (Jalaludin et al. 2004; Roemer et al. 1993; Romieu et al. 1996, 1997; Vedal et al. 1998); 35 studies used a variable “lower respiratory symptoms” or “asthma symptoms,” which in most studies (including PEACE studies) consisted of wheezing, shortness of breath, and asthma attacks (Gielen et al. 1997; Ostro et al. 2001; Pope and Dockery 1992; Roemer et al. 1998b; van der Zee et al. 1999). Other studies also included chest tightness (Delfino et al. 1998, 2002, 2003; Mortimer et al. 2002; Yu et al. 2000), sputum production (Delfino et al. 2002, 2003), or cough (Delfino et al. 1998, 2002, 2003; Mortimer et al. 2002; Ostro et al. 2001; Pope and Dockery 1992; Yu et al. 2000). In the latter studies, no separate effect estimate for cough was given except by Pope and Dockery (1992). Cough was not more precisely defined except for nocturnal cough (Just et al. 2002), cough during the day or the previous night (Peters et al. 1997), and wet and dry cough (Pope and Dockery 1992).

**Figure 1:**
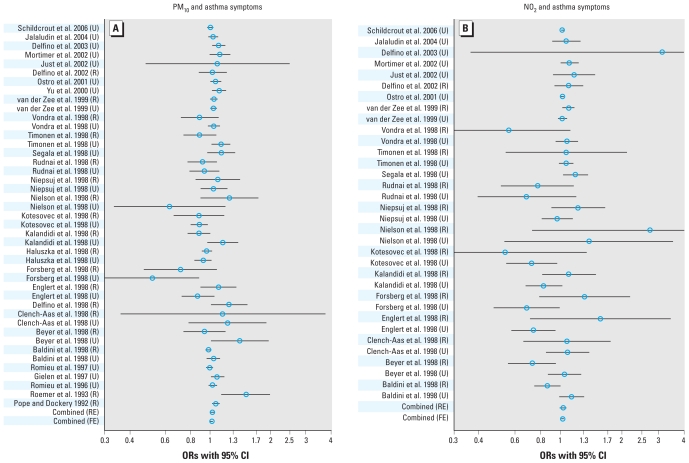
ORs with 95% CIs for the association between a rise of 10 μg/m^3^ PM_10_ (*A*) or NO_2_ (*B*) and the occurrence of asthma symptoms. Abbreviations: FE, fixed effects; R, rural; RE, random effects; U, urban.

**Figure 2:**
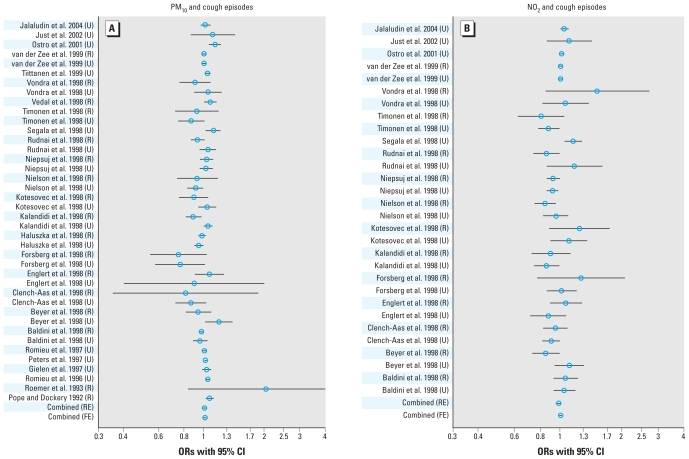
ORs with 95% CIs for the association between a rise of 10 μg/m^3^ PM_10_ (*A*) or NO_2_ (*B*) and the occurrence of cough episodes. Abbreviations: FE, fixed effects; R, rural; RE, random effects; U, urban.

**Figure 3:**
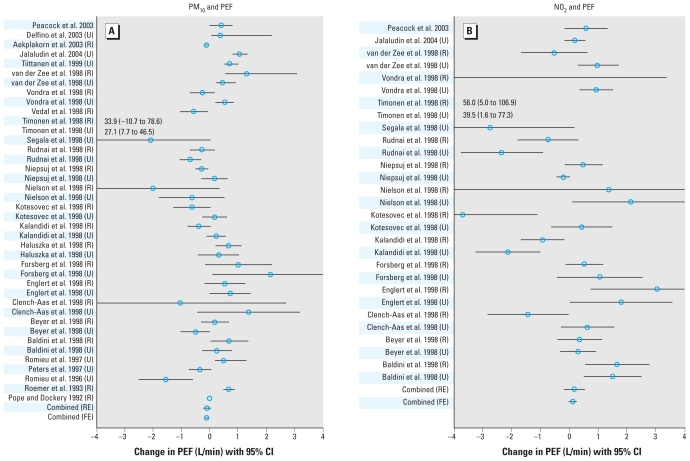
Mean increase in PEF (L/min) with 95% CIs for a rise of 10 μg/m^3^ PM_10_ (*A*) or NO_2_ (*B*). Abbreviations: FE, fixed effects; R, rural; RE, random effects; U, urban.

The effect estimates extracted from the individual studies are given in the Supplemental Material, Table 1 (doi:10.1289/ehp.0900844) and are illustrated in Figures 1–3, which also give the combined effects calculated in the meta-analysis. When we considered all the studies in the fixed-effects models, we found a significant increase of 2.3% in asthma symptoms, 1.4% for cough, and −0.117 min/L for PEF for a 10-μg/m^3^ increase in PM_10_ (Table 2). However, we observed a considerable degree of heterogeneity among the studies, with *I*^2^ ranging from 35% to 77%. Therefore, the estimates based on the random effects model are likely to represent the overall effect more accurately. For an increase of 10 μg/m^3^ of PM_10_, we found a significant increase of 2.8% in asthma symptoms, and an increase for cough (1.2%) and a decrease of PEF (−0.082 L/min) that were borderline significant. For an increase of 10 μg/m^3^ NO_2_, we found a significant increase in asthma symptoms of 3.1%. We found no clear association of NO_2_ with cough or PEF; only when we excluded the PEACE studies did we find evidence of effect for NO_2_ on cough.

When we considered all the studies, we found no evidence of publication bias. When we excluded the PEACE studies, publication bias was present for asthma symptoms for PM_10_ and NO_2_; after applying the trim-and-fill procedure, the random-effects estimates decreased from 5.5% to 3.5% and from 3.9 to 3.2, respectively, and were therefore similar to the estimates for all studies. We also saw a tendency for a similar publication bias for cough (PM_10_ and NO_2_), with significant values for the Egger test but not for the Begg test. However, the resulting trim-and-fill estimates for cough were more similar to those of the non-PEACE studies than to that for all studies (Table 2).

We found an effect modification of the effect of PM_10_ on asthma symptoms by continent (stronger association outside Europe), season (stronger association in studies carried out in summer only), study population (stronger effect among asthmatic children), and PM_10_ level (stronger association at levels < 40 μg/m^3^) (Table 3). When we excluded the PEACE studies, only season remained near significance (*p* < 0.1). For the effect of PM_10_ on cough (Table 4), there were higher associations in studies conducted outside of Europe, with lag > 2 days, or with higher NO_2_ levels; these effect modifications remained when excluding the PEACE studies. For the effect of PM_10_ on PEF (Table 4), there was a tendency for a higher decrease in PEF in asthmatic than in symptomatic children. We found no consistent effect modification, and there was no evidence for effect modification of the association between NO_2_ and any of the investigated outcomes s (Table 3 for asthma; for cough and PEF, data not shown).

The results of the sensitivity analyses based on the predefined lag 0–1 (i.e., lag 1 or 0 or 0–1) and on evening PEF showed mostly a similar pattern, especially for PM_10_, although the associations were generally weaker [see Supplemental Material, Table 3 (doi:10.1289/ehp.0900844)]. However, the associations of NO_2_ with asthma symptoms and cough were not significant in this analysis. We found effect modification even when we omitted the PEACE studies (see Supplemental Material, Tables 4 and 5), for the effect of NO_2_ on asthma symptoms, with higher associations for asthmatics and during the summer (the latter based on two studies in one stratum). Furthermore, the estimated effects of PM_10_ on asthma symptoms were higher at higher concentrations of NO_2_.

## Discussion

Our meta-analysis shows effects of PM_10_ on both asthma symptoms and cough. We found no indication of publication bias when we considered all the evidence. For NO_2_, we found statistically significant associations with asthma symptoms in the overall analysis but not in the sensitivity analysis restricted to the 0–1 lags. The effects of air pollutants on PEF were limited to PM_10_, and we saw a stronger association when we excluded the PEACE studies from the analysis. We found an indication of effect modification of PM_10_, with higher associations with asthma symptoms during summer and with cough for studies conducted outside of Europe, for a lag > 2 days, and at higher ambient NO_2_ concentrations. When considering lags 0–1 only, the pattern of effect modification was different.

A previous meta-analysis considered panel studies in children and summarized the evidence for PM_10_ up through June 2002 (Ward and Ayres 2004). Our meta-analysis extends this work further up through July 2008, adding 11 studies. On the other hand, we did not include nine studies (two from Europe) included in the Ward and Ayres (2004) analysis because the panels evaluated asymptomatic children and we focused specifically on children with asthma. Our estimates of the PM_10_ effect on asthma symptoms and cough are similar to those of the previous meta-analysis [1.04 and 1.028 for asthma symptoms, 1.04 and 1.031 for cough in Ward and Ayres (2004) and in our analysis, respectively]. Our random effects estimate for PEF is weaker than that from Ward and Ayres (−0.082 vs. −0.33 L/min for a 10-μg/m^3^ increase), whereas the fixed effects estimates are similar (−0.117 vs. −0.12 L/min).

We found no publication bias when considering all studies. However, excluding the PEACE studies, which highly influenced the estimates from the meta-analyses, resulted in clear publication bias for asthma symptoms, but less so for cough. The PEACE studies reported, on average, no effects of air pollution, with very few individual centers showing an association with PM_10_ (Roemer et al. 1998a). It is, on the one hand, the only multicenter series of studies that has been conducted with a unified protocol and whose results are not biased by publication procedures. On the other hand, limitations of the PEACE study have to be considered (Roemer et al. 1998a, 2000). There is concern that the entire study series might have been influenced by an influenza epidemic during the study period. If the study period is relatively short (e.g., 2 months as in the PEACE study), such unexpected events might confound the results, and it is generally more difficult to adjust adequately for time trend. In our analyses, we found no significant difference between studies with durations longer or shorter than 2 months. Nevertheless, for asthma symptoms, the estimate from the studies with durations longer than 2 months was slightly higher and statistically significant. In the Netherlands, where the data was collected during three winters instead of just one, there were clear effects of air pollution in symptomatic children (Roemer et al. 2000; van der Zee et al. 1999). In addition, all PEACE studies were carried out in the winter, when the effect of respiratory infections will putatively be greater compared with summer. Furthermore, in our analysis, we have found statistically greater associations in summer for asthma symptoms.

To the best of our knowledge, this is the first meta-analysis for effects related to monitored outdoor NO_2_ on respiratory health in asthmatic children, although the main investigations on NO_2_ have been extensively reviewed (U.S. EPA 2008b; WHO Regional Office for Europe 2006). *In vitro* studies at comparatively low concentrations of NO_2_, but still notably higher than ambient levels (400 ppb or 760 μg/m^3^), have shown cell damage accompanied by release of cytokines, such as tumor necrosis factor-α and interleukin-8 (Devalia et al. 1993). In controlled human studies, the same concentration for 1 hr led to an increased early and late asthmatic response (measured by forced expiratory volume in 1 sec) after challenge with house dust mite allergen compared with ordinary air (Tunnicliffe et al. 1994). Similarly, a 30-min exposure to 500 μg/m^3^ NO_2_ increased the early-phase response to an otherwise nonsymptomatic allergen dose (Strand et al. 1998). Although such concentrations can be reached during some episodes, the usual ambient concentrations of NO_2_ are lower. On the other hand, several studies on hospital admissions and emergency department visits for asthma conducted in Europe and elsewhere [reviewed by U.S. EPA (2008b); WHO Regional Office for Europe (2006)] did find an independent effect of NO_2_. Therefore, the extent to which the observed associations are related to a direct effect of NO_2_ and/or reflect the fact that NO_2_ is a marker for the urban pollution mix, particularly for ultrafine particles PM (Seaton and Dennekamp 2003), remains to be investigated. The correlation between PM_10_ and NO_2_ varies across settings (Katsouyanni et al. 2001), with the pollution mix related to NO_2_ generally being more variable in space and time. Notwithstanding these differences, the estimated effect size for NO_2_ observed in this meta-analysis is similar to that of the PM_10_ component, except for PEF.

There may be a concern that bias might be introduced when selecting effects that were not for the same lag. Our additional analysis for lags 0–1 provided nonsignificant estimates for NO_2_ but significant associations with PM_10_. It remains to be shown whether such a short lag is the most adequate for measuring the effect, given that higher associations may be observed at longer lags, as we found in our analysis of effect modification. Unfortunately, longer lags are less consistently reported in the literature.

There are limitations of the panel studies we have considered. When evaluating symptoms, the possibility of a confounding role of medications should be considered. Medication use on polluted days may influence symptoms and lung function. Although the PEACE studies found no correlation between the number of children using asthma medication and air pollution levels (Roemer et al. 2000), this does not account for the possibility that asthmatic children increase the dose on such days. Information regarding this possibility is generally missing in the individual study reports. The evaluation of the effect on PEF is difficult because of the large between-individual variability of this indicator that is likely to be strongly influenced by medication use among diseased subjects. Finally, another difficulty is that the measured pollutants are only part of a more complex air pollution mixture, and the effects of “PM_10_” and “NO_2_” may vary among studies and may be a less or more adequate measure of the effects of air pollution. In a meta-analysis, it is not possible to adequately assess the problems related to these mixes. Multipollutant (mostly two-pollutant) models were calculated for only 10 of the study populations, and the combinations of the pollutants varied among studies. Only if the raw data were available for all studies could one attempt to tease out individual pollutant effects and also avoid overestimation of the individual effect. It will nonetheless be a daunting task, because in most cases criteria air pollutants are measured, which may be indicators of different unmeasured compounds in different areas. Delfino et al. (2003) reported, for example, that the effect of “PM_10_” was lower when, for example, organic carbon, benzene, or *m*,*p*-xylene was included in two-pollutant models. This may be a general finding, or it may be typical for the region investigated. The results presented here therefore are not to be strictly understood as the effect of PM_10_ only or NO_2_ only; the greater context must be borne in mind.

We observed a high degree of heterogeneity among the investigated studies. Stratifying by the identified effect modifiers reduced the heterogeneity only to some extent. We obtained the greatest reduction in heterogeneity when using the same lag for all studies. Sources of heterogeneity may be linked to various design aspects of the study, such as the inclusion criteria for the panel, duration of the study, and the analytical strategies. For the PEACE study with its standardized study protocol and common analytical strategy, we calculated an *I*^2^ ranging from 40% to 79% depending on the outcome/pollutant only for the analysis using different lags, whereas the analyses with the uniform shorter lag reduced the heterogeneity among PEACE studies for symptoms and PEF (data not shown). Although this may highlight the importance of a standardized study protocol, caution is needed until it is better known which lag is the most appropriate. Therefore, other potential sources of the observed heterogeneity, such as differences in the air pollution mix related to spatial or temporal variability, may still be of importance even in well- standardized studies. Different baseline characteristics of the populations studied may also have their influence.

The estimated effect of PM_10_ on asthma was higher in studies that were conducted in the summer. The composition of the air pollution mix may also be the reason for higher observed effects of PM_10_ in studies that have been conducted in summer only. Summer pollution is qualitatively different from winter pollution: O_3_ levels are higher, and in general the air pollution mixture is more strongly influenced by photochemical reaction. Ward and Ayres (2004) observed in their analysis a higher estimated effect in studies conducted in periods of high O_3_ levels. A time-series analysis of Atkinson et al. (2001) observed effect modification by O_3_ for hospital admission for respiratory conditions in persons older than 65 years, although not for asthma admissions in children or adults. Alternative reasons could be that the PM_10_ effect is confounded by the effect of O_3._ However, independent effects have been found for PM_2.5_, and for PM_2.5–10_ concerning cough [for a more detailed discussion, see Ward and Ayres (2004)]. The higher estimated effect of PM_10_ in the summer could also be linked to more (active) time spent outside, which could act in several ways. First, it would reduce misclassification due to less exposure to indoor conditions. Second, it could increase the effect of PM_10_ through increased inhalation during the activities outside (e.g., exercise), which also could increase the effect of O_3_.

Consideration of longer lags did result in elevated associations of PM_10_ with cough. This seems plausible because air pollution may act not only as a short-term trigger but also as a priming event by inducing processes of enhanced airways inflammation (Kimber 1998) that will build up over a period of hours to days and result in subsequent bronchial hyperreactivity (Mortimer et al. 2002). Indeed, lengthy lag periods have been found in panel studies as well as time-series studies of emergency department visits (Halonen et al. 2008; Mortimer et al. 2002).

Continent modified the association of PM_10_ with cough; we found a significant combined effect only for the studies outside of Europe, whereas for the European studies the combined effect was null (OR = 0.998; 95% CI, 0.983–1.014). This estimate is similar to that reported by Anderson et al. (2004) for Europe (OR = 0.999; 95% CI, 0.987–1.011). At first glance, a similar effect modification was present for asthma symptoms, but this disappeared after exclusion of the PEACE studies. It therefore remains speculative whether this is really an effect for Europe or is attributable to some other characteristic that is specifically related to the PEACE study.

Nevertheless, a stronger association of PM_10_ with respiratory symptoms reported in the United States compared with Europe was also observed in an earlier meta-analysis, conducted before the PEACE study, that also included healthy children (Dockery and Pope 1994). One plausible explanation could be different pollutant mixes on the two continents. The extent to which these differences are systematic and will provide relevant information remains to be investigated, given that also within the United States and within Europe there are marked differences concerning the air pollution mix, which may result in differing health effects via effect modification or due to a different composition of PM_10_ (Katsouyanni et al. 2001; Levy et al. 2000).

In our analysis, we found the association of PM_10_ with cough to be stronger for higher ambient NO_2_ concentration. However, we did not see this effect in the analysis restricted to lags 0–1, but in this latter analysis we found higher associations at higher NO_2_ levels with asthma symptoms. Effect modification by NO_2_ has been found in time series studies on mortality in Europe (Katsouyanni et al. 2001), and to a lesser extent in the United States (Levy et al. 2000). It has been discussed that NO_2_ is a marker for a certain air pollution mixture, notably arising from traffic, which is more noxious for health.

## Conclusion

Our meta-analysis provides strong evidence for an effect of PM_10_ as an aggravating factor of asthma in children. Although there is no firm toxicologic evidence of adverse health effects of NO_2_ at ambient levels to date, the epidemiologic results suggest an adverse effect of NO_2_ on respiratory health in children with asthma. However, caution is needed in the final conclusion for NO_2_ because the association with asthma attacks was not robust to lag specification. The finding may reflect the fact that NO_2_ is associated at extended lags, or it may be only an artifact due to our method of choosing the specific lag to be included in the meta-analysis. More consistent reporting of longer lags is needed in panel studies to better judge the effect of monitored outdoor NO_2_. The results of the study support the need to protect asthmatic children with strict air quality standards for PM_10_ and, considering the precautionary principle, also for NO_2_.

## Figures and Tables

**Table 1:** Study characteristics of the panel studies.

Study	Outcomes studied^a^	Pollutant studied	Year of study	Continent	Urban/rural	*n* (duration in days)	Season	Population	Pollutant 24-hr mean (μg/m^3^)^b^	
									PM_10_	NO_2_
Pope and Dockery 1992	LRS, cough, PEF	PM_10_	1990	Other	Rural	39 (70)	Other	Symptomatics	56	—
Roemer et al. 1993	LRS, cough, PEF	PM_10_	1990	Europe	Rural	73 (90)	Other	Symptomatics	76	71^c^
Romieu et al. 1996	LRS, cough, PEF	PM_10_	1991	Other	Urban	71 (60)	Other	Asthmatics	167	75
Gielen et al. 1997	LRS, cough	PM_10_	1995	Europe	Urban	61 (60)	Summer only	Asthmatics	31	—
Peters et al. 1997	Cough, PEF	PM_10_	1991	Europe	Urban	89 (210)	Other	Asthmatics	55	—
Romieu et al. 1997	LRS, cough, PEF	PM_10_	1991	Other	Urban	67 (60)	Other	Asthmatics	54	37–169^d^
Delfino et al. 1998	LRS	PM_10_	1995	Other	Rural	24 (90)	Summer only	Asthmatics	43	—
Segala et al. 1998	LRS, cough, PEF	PM_10_, NO_2_	1992	Europe	Urban	41 (175)	Other	Asthmatics	34	57
Vedal et al. 1998	Cough, PEF	PM_10_	1990	Other	Rural	75 (492)	Other	Asthmatics	27	—
Tiittanen et al. 1999	Cough, PEF	PM_10_	1995	Europe	Urban	49 (42)	Other	Symptomatics	50%ile, 28	50%ile, 15
van der Zee et al. 1999	LRS, cough	PM_10_, NO_2_	1993	Europe	Urban	142 (90)	Other	Symptomatics	38	49
van der Zee et al. 1999	LRS, cough	PM_10_, NO_2_	1993	Europe	Rural	178 (90)	Other	Symptomatics	31	27
Jalaludin et al. 2000	PEF	PM_10_, NO_2_	1994	Other	Urban	125 (300)	Other	Asthmatics	23	28
Yu et al. 2000	LRS	PM_10_	1993	Other	Urban	133 (58)	Other	Asthmatics	10	—
Ostro et al. 2001	LRS, cough	PM_10_, NO_2_	1993	Other	Urban	138 (90)	Summer only	Asthmatics	51	77^c^
Delfino et al. 2002	LRS	PM_10_, NO_2_	1996	Other	Rural	22 (61)	Other	Asthmatics	20	26^c^
Just et al. 2002	LRS, cough	PM_10_, NO_2_	1996	Europe	Urban	82 (90)	Other	Asthmatics	24	54
Mortimer et al. 2002	LRS	PM_10_, NO_2_	1993	Other	Urban	846 (14)	Summer only	Asthmatics	—	61
Aekplakorn et al. 2003	PEF	PM_10_	1997	Other	Rural	88 (53–61)	Other	Asthmatics	50%ile, 22–25^e^	No NO_2_ measured
Delfino et al. 2003	LRS, PEF	PM_10_, NO_2_	1999	Other	Urban	22 (90)	Other	Asthmatics	60	8^c^
Peacock et al. 2003	PEF	PM_10_, NO_2_	1996	Europe	—	179 (63)	Other	Symptomatics	18–23^e^	31–36^e^
Jalaludin et al. 2004	LRS, cough	PM_10_, NO_2_	1994	Other	Urban	148 (> 30)	Other	Symptomatics	23 (0600–2100 hr)	28 (0600–2100 hr)
Schildcrout et al. 2006	LRS	PM_10_, NO_2_	1993	Other	Urban	990 (60)	Other	Asthmatics	50%ile, 18–34^e^	50%ile, 34–59^e^
PEACE studies										
Baldini et al. 1998	LRS, cough, PEF	PM_10_, NO_2_	1993	Europe	Urban	68 (65)	Other	Symptomatics	62	68
Baldini et al. 1998	LRS, cough, PEF	PM_10_, NO_2_	1993	Europe	Rural	60 (65)	Other	Symptomatics	70	33
Beyer et al. 1998	LRS, cough, PEF	PM_10_, NO_2_	1993	Europe	Urban	75 (172)	Other	Symptomatics	40	27
Beyer et al. 1998	LRS, cough, PEF	PM_10_, NO_2_	1993	Europe	Rural	63 (172)	Other	Symptomatics	33	26
Clench-Aas et al. 1998	LRS, cough, PEF	PM_10_, NO_2_	1993	Europe	Urban	56 (70)	Other	Symptomatics	19	49
Clench-Aas et al. 1998	LRS, cough, PEF	PM_10_, NO_2_	1993	Europe	Rural	68 (70)	Other	Symptomatics	11	21
Englert et al. 1998	LRS, cough, PEF	PM_10_, NO_2_	1993	Europe	Urban	50 (58)	Other	Symptomatics	52	38
Englert et al. 1998	LRS, cough, PEF	PM_10_, NO_2_	1993	Europe	Rural	66 (58)	Other	Symptomatics	43	21
Forsberg et al. 1998	LRS, cough, PEF	PM_10_, NO_2_	1993	Europe	Urban	75 (84)	Other	Symptomatics	13	25
Forsberg et al. 1998	LRS, cough, PEF	PM_10_, NO_2_	1993	Europe	Rural	72 (84)	Other	Symptomatics	12	15
Haluszka et al. 1998	LRS, cough, PEF	PM_10_	1993	Europe	Urban	73 (82)	Other	Symptomatics	60	—
Haluszka et al. 1998	LRS, cough, PEF	PM_10_	1993	Europe	Rural	76 (76)	Other	Symptomatics	56	—
Kalandidi et al. 1998	LRS, cough, PEF	PM_10_, NO_2_	1993	Europe	Urban	87 (60)	Other	Symptomatics	99	75
Kalandidi et al. 1998	LRS, cough, PEF	PM_10_, NO_2_	1993	Europe	Rural	80 (60)	Other	Symptomatics	50	20
Kotesovec et al. 1998	LRS, cough, PEF	PM_10_, NO_2_	1993	Europe	Urban	91 (60)	Other	Symptomatics	74	49
Kotesovec et al. 1998	LRS, cough, PEF	PM_10_, NO_2_	1993	Europe	Rural	77 (60)	Other	Symptomatics	32	13
Nielsen et al. 1998	LRS, cough, PEF	PM_10_, NO_2_	1993	Europe	Urban	78 (60)	Other	Symptomatics	23	21
Nielsen et al. 1998	LRS, cough, PEF	PM_10_, NO_2_	1993	Europe	Rural	82 (60)	Other	Symptomatics	16	9
Niepsuj et al. 1998	LRS, cough, PEF	PM_10_, NO_2_	1993	Europe	Urban	72 (83)	Other	Symptomatics	69	69
Niepsuj et al. 1998	LRS, cough, PEF	PM_10_, NO_2_	1993	Europe	Rural	73 (83)	Other	Symptomatics	74	70
Rudnai et al. 1998	LRS, cough, PEF	PM_10_, NO_2_	1993	Europe	Urban	76 (61)	Other	Symptomatics	61	35
Rudnai et al. 1998	LRS, cough, PEF	PM_10_, NO_2_	1993	Europe	Rural	63 (67)	Other	Symptomatics	52	25
Timonen et al. 1998	LRS, cough, PEF	PM_10_, NO_2_	1993	Europe	Urban	85 (72)	Other	Symptomatics	18	28
Timonen et al. 1998	LRS, cough, PEF	PM_10_, NO_2_	1993	Europe	Rural	84 (72)	Other	Symptomatics	13	14
Vondra et al. 1998	LRS, cough, PEF	PM_10_, NO_2_	1993	Europe	Urban	66 (85)	Other	Symptomatics	53	45
Vondra et al. 1998	LRS, cough, PEF	PM_10_, NO_2_	1993	Europe	Rural	68 (85)	Other	Symptomatics	50	13
van der Zee et al. 1998	PEF	PM_10_, NO_2_	1993	Europe	Urban	55 (101)	Other	Symptomatics	45	46
van der Zee et al. 1998	PEF	PM_10_, NO_2_	1993	Europe	Rural	71 (93)	Other	Symptomatics	44	27

a LRS is equivalent to asthma symptoms.

b Mean of the 24-hr means unless otherwise indicated.

c Extrapolated from 1-hr maximum.

d Range of means over the study period.

e Means from more than one location.

**Table 2:** Association of PM_10_ and NO_2_ exposure with episodes of asthma symptoms, episodes of cough, and PEF in children symptomatic for or diagnosed with asthma.

	PM_10_					NO_2_				
Symptom	*n*	OR_F_/β_F_ (95% CI)	OR_R_/β_R_ (95% CI)	*p*-Value(*I*^2^)	*p*-Value^a^	*n*	OR_F_/β_F_ (95% CI)	OR_R_/β_R_ (95% CI)	*p*-Value(*I*^2^)	*p*-Value^a^
Asthma symptoms										
All studies	43	1.023 (1.013 to 1.034)	1.028 (1.006 to 1.051)	< 0.001 (59%)	0.779 (0.675)	34	1.026 (1.016 to 1.037)	1.031 (1.001 to 1.062)	< 0.001 (50%)	0.746 (0.594)
Without PEACE studies	17	1.035 (1.023 to 1.047)	1.055 (1.032 to 1.078)	0.002 (56%)	0.000 (0.053)	10	1.028 (1.017 to 1.039)	1.039 (1.018 to 1.061)	0.125 (35%)	0.001 (0.152)
Trim-and-fill estimate	24	1.028 (1.016 to 1.039)	1.035 (1.012 to 1.058)	< 0.0001 (61%)		15	1.026 (1.015 to 1.037)	1.032 (1.008 to 1.057)	0.052 (41%)	

Cough										
All studies	40	1.014 (1.008 to 1.019)	1.012 (0.997 to 1.026)	< 0.001 (69%)	0.442 (0.316)	30	1.006 (0.995 to 1.016)	0.987 (0.960 to 1.014)	< 0.001 (65%)	0.394 (0.158)
Without PEACE studies	14	1.020 (1.014 to 1.026)	1.035 (1.020 to 1.050)	< 0.001 (72%)	0.002 (0.07)	6	1.018 (1.006 to 1.030)	1.031 (1.005 to 1.057)	0.006 (69%)	0.007 (0.085)
Trim-and-fill estimate	19	1.018 (1.012 to 1.024)	1.027 (1.011 to 1.043)	< 0.001 (72%)		8	1.015 (1.003 to 1.026)	1.018 (0.988 to 1.050)	< 0.001 (76%)	

PEF^b^										
All studies	40	−0.117 (−0.160 to −0.073)	−0.082 (−0.214 to 0.050)	< 0.001 (72%)	0.456 (0.428)	29	0.130 (−0.008 to 0.268)	0.180 (−0.184 to 0.544)	< 0.001 (77%)	0.433 (0.925)
Without PEACE studies	12	−0.145 (−0.195 to −0.096)	−0.272 (−0.449 to −0.095)	< 0.001 (69%)	0.061 (0.451)	3	0.232 (−0.091 to 0.556)	0.170 (−0.590 to 0.929)	0.088 (59%)	0.594 (1.000)

Abbreviations: OR_F_/β_F_ and OR_R_/β_R_, combined estimate of the OR (or regression coefficient β for PEF in L/min) from the fixed-effects and random-effects models, respectively, for a 10-μg/m^3^ increase of pollutant; *p*(*I*^2^), *p*-value for test of heterogeneity based on Cochrane’s *Q*, with *I*^2^ of Higgins and Thompson reflecting the proportion of total variation in the estimate that is due to heterogeneity between studies.

a *p*-Value for Egger (Begg) bias test.

b The metatrim command in STATA did not perform any trimming for this outcome ( “no trimming performed, data unchanged”).

**Table 3:** Stratum-specific combined estimates of the association of PM_10_ and NO_2_ exposure with episodes of wheezing in children symptomatic for or diagnosed with asthma.

	PM_10_						NO_2_					
	All studies			PEACE studies excluded			All studies			PEACE studies excluded		
Stratum	*n*	OR_R_ (95% CI)	*p*_Strata_*p*_het_ (*I*^2^)	*n*	OR_R_ (95% CI)	*p*_Strata_*p*_het_ (*I*^2^)	*n*	OR_R_ (95% CI)	*p*_Strata_*p*_het_ (*I*^2^)	*n*	OR_R_ (95% CI)	*p*_Strata_*p*_het_ (*I*^2^)
Continent			0.066			0.457			0.366			0.084

Europe	32	1.008 (0.975–1.043)	< 0.001 (60%)	6	1.069 (1.025–1.116)	0.121 (43%)	28	0.998 (0.942–1.058)	< 0.001 (56%)	4	1.085 (1.019–1.155)	0.126 (47%)
Other	11	1.050 (1.022–1.077)	0.006 (60%)	11	1.050 (1.022–1.077)	0.006 (59%)	6	1.025 (1.014–1.036)	0.471 (0%)	6	1.025 (1.014–1.036)	0.471 (0%)

Season			0.006			0.095			0.332			0.920

Summer only	5	1.090 (1.045–1.136)	0.682 (0%)	5	1.090 (1.045–1.136)	0.682 (0%)	3	1.057 (0.987–1.133)	0.166 (44%)	3	1.057 (0.987–1.133)	0.166 (44%)
Other	38	1.020 (0.997–1.043)	< 0.001 (60%)	12	1.046 (1.022–1.071)	0.003 (61%)	31	1.016 (0.974–1.059)	< 0.001 (52%)	7	1.053 (1.015–1.092)	0.112 (42%)

Population			0.029			0.963			0.132			0.434

Asthmatics	12	1.056 (1.025–1.088)	0.009 (56%)	12	1.056 (1.025–1.088)	0.009 (56%)	7	1.034 (1.011–1.059)	0.132 (39%)	7	1.034 (1.011–1.059)	0.132 (39%)
Symptomatics	31	1.007 (0.976–1.039)	< 0.001 (62%)	5	1.055 (1.023–1.088)	0.107 (47%)	27	0.986 (0.931–1.045)	< 0.001 (54%)	3	1.056 (1.010–1.104)	0.299 (17%)

Duration			0.758			0.645			0.285			0.192

≤ 2 months	14	1.022 (0.978–1.068)	0.001 (63%)	6	1.049 (1.013–1.087)	0.069 (51%)	10	0.954 (0.819–1.110)	0.003 (64%)	2	1.098 (1.009–1.194)	0.698 (0%)
> 2 months	29	1.031 (1.005–1.058)	< 0.001 (59%)	11	1.061 (1.029–1.094)	0.003 (62%)	24	1.037 (1.009–1.066)	0.011 (44%)	8	1.036 (1.014–1.057)	0.121 (39%)

Lag			0.325			0.438			0.601			0.597

≤ 2 days	22	1.020 (0.994–1.046)	< 0.001 (64%)	11	1.047 (1.020–1.076)	0.021 (53%)	16	1.016 (0.966–1.069)	0.002 (58%)	6	1.043 (1.004–1.084)	0.190 (33%)
> 2 days	21	1.044 (1.005–1.084)	0.012 (46%)	6	1.066 (1.028–1.106)	0.072 (51%)	18	1.037 (0.981–1.096)	0.020 (45%)	4	1.061 (1.009–1.115)	0.098 (52%)

PM_10_ level			0.102			0.795			0.079			0.612

< 40 μg/m^3^	19	1.057 (1.020–1.095)	0.053 (37%)	9	1.057 (1.034–1.079)	0.565 (0%)	16	1.062 (1.005–1.121)	0.064 (38%)	6	1.074 (1.029–1.121)	0.319 (15%)
≥ 40 μg/m^3^	23	1.016 (0.985–1.048)	< 0.001 (65%)	7	1.063 (1.021–1.106)	0.007 (66%)	17	0.982 (0.918–1.050)	0.001 (58%)	3	1.051 (0.976–1.131)	0.179 (42%)

NO_2_ level			0.201			0.763			0.116			0.280

< 40 μg/m^3^	22	1.007 (0.966–1.051)	< 0.001 (59%)	5	1.059 (1.031–1.087)	0.586 (0%)	21	0.972 (0.894–1.056)	0.002 (54%)	4	1.095 (1.034–1.159)	0.798 (0%)
≥ 40 μg/m^3^	15	1.042 (1.010–1.076)	0.018 (49%)	8	1.051 (1.013–1.091)	0.038 (53%)	12	1.048 (1.002–1.097)	0.023 (50%)	5	1.053 (1.009–1.098)	0.100 (49%)

Rural/urban			0.261			0.289			0.559			0.052

Rural	18	1.008 (0.965–1.053)	< 0.001 (61%)	5	1.082 (1.022–1.145)	0.058 (56%)	14	0.997 (0.887–1.122)	0.008 (54%)	2	1.098 (1.033–1.167)	0.981 (0%)
Urban	25	1.038 (1.012–1.064)	< 0.001 (59%)	12	1.047 (1.023–1.071)	0.021 (51%)	20	1.033 (1.004–1.063)	0.007 (49%)	8	1.030 (1.012–1.049)	0.226 (25%)

Abbreviations: OR_R_, combined estimate of the OR from the random effects model for 10-μg/m3 increase in pollutant; *p*_Strata_*p*_het_ (*I*^2^), *p*-value for differences between strata and *p*-value for test of heterogeneity based on Cochrane’s *Q*, with *I*^2^ of Higgins and Thompson reflecting the proportion of total variation in the estimate that is due to heterogeneity between studies.

**Table 4:** Stratum-specific combined estimates of the association of PM_10_ exposure with change in PEF (L/min) and with cough episodes in children symptomatic for or diagnosed with asthma.

	PEF						Cough					
	All studies			PEACE studies excluded			All studies			PEACE studies excluded		
Stratum	*n*	β_R_ (95% CI)	*p*_Strata_*p*_het_ (*I*^2^)	*n*	β_R_ (95% CI)	*p*_Strata_*p*_het_ (*I*^2^)	*n*	OR_R_ (95% CI)	*p*_Strata_*p*_het_ (*I*^2^)	*n*	OR_R_ (95% CI)	*p*_Strata_*p*_het_ (*I*^2^)
Continent			0.041			0.750			0.001			0.047

Europe	33	0.002 (−0.182 to 0.186)	< 0.001 (72%)	5	−0.235 (−0.600 to 0.131)	0.006 (73%)	34	0.998 (0.983 to 1.014)	< 0.001 (62%)	8	1.020 (1.006 to 1.034)	0.026 (56%)
Other	7	−0.305 (−0.534 to −0.076)	0.003 (69%)	7	−0.305 (−0.534 to −0.076)	0.003 (69%)	6	1.053 (1.024 to 1.082)	0.004 (71%)	6	1.053 (1.024 to 1.082)	0.004 (71%)

Season									0.260			0.905

Summer only							2	1.039 (0.992 to 1.088)	0.602 (0%)	2	1.039 (0.992 to 1.088)	0.602 (0%)
Other	40	−0.082 (−0.214 to 0.050)	< 0.001 (72%)	12	−0.272 (−0.449 to −0.095)	< 0.001 (69%)	38	1.010 (0.996 to 1.025)	< 0.001 (70%)	12	1.035 (1.019 to 1.051)	< 0.001 (76%)

Population			0.007			0.086			0.001			0.217

Asthmatics	7	−0.549 (−0.920 to −0.177)	0.006 (67%)	7	−0.549 (−0.920 to −0.177)	0.006 (67%)	8	1.046 (1.022 to 1.071)	0.001 (70%)	8	1.046 (1.022 to 1.071)	0.001 (70%)
Symptomatics	33	0.010 (−0.159 to 0.180)	< 0.001 (73%)	5	−0.148 (−0.415 to 0.119)	0.002 (76%)	32	0.995 (0.978 to 1.013)	< 0.001 (63%)	6	1.026 (1.006 to 1.046)	0.005 (70%)

Duration			0.402			0.416			0.422			0.762

≤ 2 months	12	−0.161 (−0.394 to 0.071)	< 0.001 (67%)	4	−0.440 (−0.843 to −0.037)	0.010 (73%)	13	1.019 (0.995 to 1.043)	0.003 (59%)	5	1.034 (1.017 to 1.051)	0.188 (35%)
> 2 months	28	−0.032 (−0.225 to 0.160)	< 0.001 (74%)	8	−0.241 (−0.500 to 0.018)	0.079 (69%)	27	1.007 (0.990 to 1.026)	< 0.001 (70%)	9	1.038 (1.017 to 1.059)	< 0.001 (77%)

Lag			0.325			0.189			0.018			0.030

≤ 2 days	14	−0.167 (−0.354 to 0.021)	< 0.001 (70%)	8	−0.203 (−0.426 to 0.020)	0.001 (71%)	19	0.997 (0.979 to 1.014)	< 0.001 (74%)	6	1.022 (1.006 to 1.038)	0.004 (71%)
> 2 days	26	−0.025 (−0.237 to 0.187)	< 0.001 (73%)	4	−0.396 (−0.578 to −0.214)	0.392 (0%)	21	1.036 (1.009 to 1.065)	0.001 (56%)	8	1.067 (1.030 to 1.106)	0.001 (71%)

PM_10_ level			0.774			0.344			0.706			0.173

< 40 μg/m^3^	14	−0.021 (−0.441 to 0.398)	< 0.001 (68%)	4	−0.116 (−0.613 to 0.381)	0.006 (76%)	17	1.006 (0.983 to 1.029)	0.002 (57%)	7	1.022 (1.004 to 1.041)	0.047 (53%)
≥ 40 μg/m^3^	25	−0.086 (−0.233 to 0.061)	< 0.001 (74%)	7	−0.380 (−0.607 to −0.152)	0.005 (68%)	22	1.012 (0.991 to 1.033)	< 0.001 (74%)	6	1.045 (1.018 to 1.073)	< 0.001 (79%)

NO_2_ level			0.722			0.028			0.012			0.031

< 40 μg/m^3^	21	−0.018 (−0.278 to 0.242)	68%*	3	0.144 (−0.224 to 0.512)	0.155 (46%)	20	0.980 (0.954 to 1.007)	< 0.001 (60%)	3	1.013 (1.001 to 1.025)	0.342 (7%)
≥ 40 μg/m^3^	11	−0.091 (−0.399 to 0.216)	80%*	3	−1.085 (−2.120 to −0.051)	0.028 (72%)	13	1.032 (1.001 to 1.064)	< 0.001 (72%)	6	1.065 (1.019 to 1.113)	< 0.001 (82%)

Rural/urban			0.911			0.433			0.116			0.604

Rural	18	−0.125 (−0.286 to 0.036)	< 0.001 (65%)	4	−0.301 (−0.507 to −0.096)	0.020 (70%)	17	0.994 (0.968 to 1.021)	< 0.001 (65%)	4	1.050 (0.995 to 1.109)	0.003 (79%)
Urban	21	−0.108 (−0.360 to 0.144)	< 0.001 (75%)	7	−0.473 (−0.851 to −0.095)	0.008 (66%)	23	1.020 (1.002 to 1.039)	< 0.001 (69%)	10	1.035 (1.017 to 1.052)	< 0.001 (70%)

Abbreviations: OR_R_/β_R_, combined estimate of the OR (or regression coefficient β for PEF in L/min) from the random effects model for a 10-μg/m increase in pollutant; *p*_Strata_*p*_het_ (*I* ), *p*-value for differences between strata and *p*-value for test of heterogeneity based on Cochrane’s *Q*, with *I*^2^ of Higgins and Thompson reflecting the proportion of total variation in the estimate that is due to heterogeneity between studies.

* *p* < 0.001.
